# Supplementary material for: Improving the Autofluorescence of Lophira alata Woody Cells via the Removal of Extractives
Source: Polymers (Basel). 2023 Aug 1;15(15):3269. doi: 10.3390/polym15153269 (PMC10422229; doi:10.3390/polym15153269)
Supplement: Supplementary file 1 [file polymers-15-03269-s001.zip › polymers-2423476-supplementary.pdf]

Supplementary Materials: Improving the Autofluorescence of *Lophira alata* Woody Cells via the Removal of Extractives

Zhaoyang Yu [1], Dongnian Xu [1], Jinbo Hu [1,2,3,*], Shanshan Chang [1,*], Gonggang Liu [1], Qiongtao Huang [2], Jin Han [1], Ting Li [3], Yuan Liu [1] and Xiaodong (Alice) Wang [4]

[1] College of Materials Science and Engineering, Central South University of Forestry and Technology, Changsha 410004, China; yuzhaoyangvip@126.com (Z.Y.); 15707483515@163.com (D.X.); liugonggang@gmail.com (G.L.); hanjin@csuft.edu.cn (J.H.); liuyuan601220@163.com (Y.L.)
[2] Department of Research and Development Center, Yihua Lifestyle Technology Co., Ltd., Shantou 515834, China; huangqt@yihua.com
[3] Hunan Taohuajiang Bamboo Science & Technology Co., Ltd., Taojiang 413400, China; liting0907thj@163.com
[4] Department of Wood and Forest Sciences, Laval University, Quebec, QC G1V 0A6, Canada; xiaodong.wang@sbf.ulaval.ca
* Correspondence: hjb1999@hotmail.com (J.H.); changelxy@hotmail.com (S.C.)

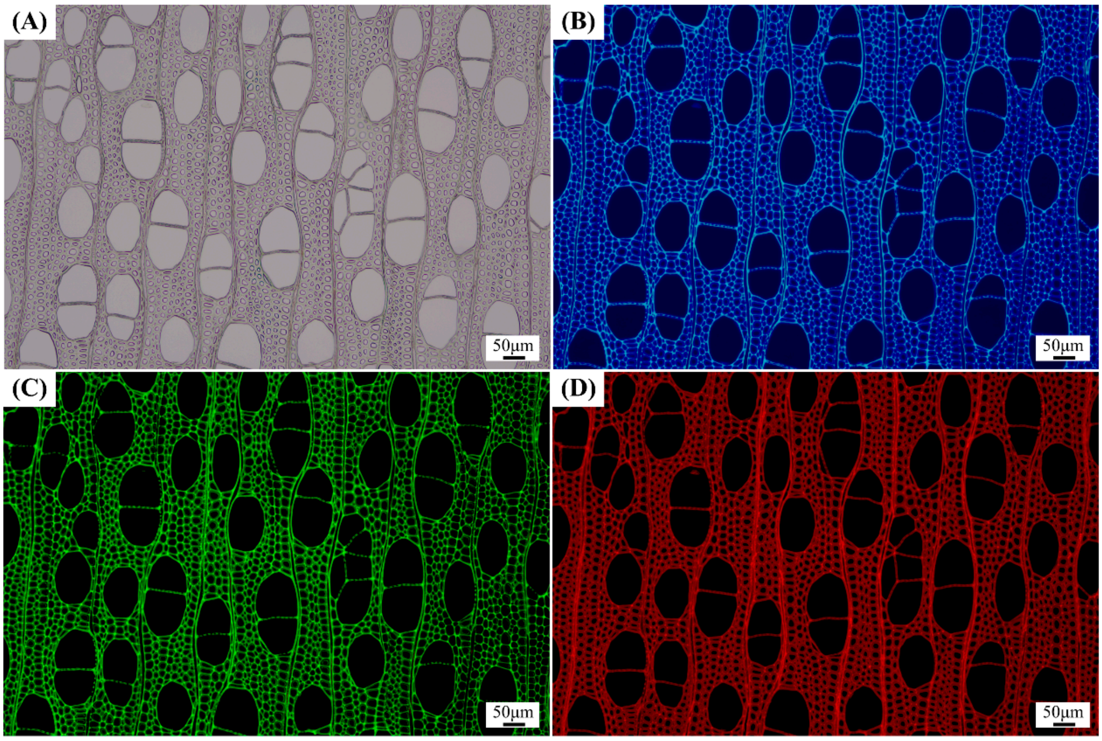

Figure S1. Microscopic images observed in bright field and fluorescence on cross section of *Populus deltoides*. Observe in visible light (A), UV light excitation (B. Emission wavelengths: 330-380 nm), blue light excitation (C. Emission wavelengths: 450-490 nm), green light excitation (D. Emission wavelengths: 510-560 nm). Scale bars = 50 μm.
